# Comparison of crystal structures of 4-(benzo[*b*]thio­phen-2-yl)-5-(3,4,5-tri­meth­oxy­phen­yl)-2*H*-1,2,3-triazole and 4-(benzo[*b*]thio­phen-2-yl)-2-methyl-5-(3,4,5-tri­meth­oxy­phen­yl)-2*H*-1,2,3-triazole

**DOI:** 10.1107/S1600536814023095

**Published:** 2014-10-24

**Authors:** Narsimha Reddy Penthala, Nikhil Reddy Madadi, Shobanbabu Bommagani, Sean Parkin, Peter A. Crooks

**Affiliations:** a Department of Pharmaceutical Sciences, College of Pharmacy, University of Arkansas for Medical Sciences, Little Rock, AR 72205, USA; b Department of Chemistry, University of Kentucky, Lexington KY 40506, USA

**Keywords:** combretastatin A-4 analog, anti-cancer agent, triazole ring, hydrogen bonding, crystal structure

## Abstract

The title compound, C_19_H_17_N_3_O_3_S (I), was prepared by a [3 + 2]cyclo­addition azide condensation reaction using sodium azide and l-proline as a Lewis base catalyst. *N*-Methyl­ation of compound (I) using CH_3_I gave compound (II), C_20_H_19_N_3_O_3_S. The benzo­thio­phene ring systems in (I) and (II) are almost planar, with r.m.s deviations from the mean plane = 0.0205 (14) in (I) and 0.016 (2) Å in (II). In (I) and (II), the triazole rings make dihedral angles of 32.68 (5) and 10.43 (8)°, respectively, with the mean planes of the benzo­thio­phene ring systems. The trimeth­oxy phenyl rings make dihedral angles with the benzo­thio­phene rings of 38.48 (4) in (I) and 60.43 (5)° in (II). In the crystal of (I), the mol­ecules are linked into chains by N—H⋯O hydrogen bonds with *R* ^2^_1_(5) ring motifs. After the *N*-methyl­ation of structure (I), no hydrogen-bonding inter­actions were observed for structure (II). The crystal structure of (II) has a minor component of disorder that corresponds to a 180° flip of the benzo­thio­phene ring system [occupancy ratio 0.9363 (14):0.0637 (14)].

## Abstract 2

In the crystal structure of (I), the mol­ecules are linked into chains by N—H⋯O hydrogen bonds with 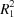
(5) ring motifs. After the *N*-methyl­ation of structure (I), no hydrogen-bonding inter­actions were observed for structure (II).

## Chemical context   

In continuation of our work on the development of benzo­thio­phene cyano combretastatin A-4 analogs as anti-cancer agents (Penthala *et al.*, 2013 ▶), we have synthesized a series of novel CA-4 analogs by constructing a triazole ring structure (I) by chemical modification of the cyano group on the stilbene unit of cyano-CA-4 analogs utilizing a [3 + 2]cyclo­addition azide condensation reaction with sodium azide in the presence of l-proline Lewis base as catalyst. This chemical modification is essential to restrict the tendency toward *cis–trans* isomerization of the cyano-stilbene moiety in cyano-CA-4 analogs (Penthala *et al.*, 2013 ▶). To further check the position of the hydrogen atom in the triazole ring system in (I), an *N*-methyl­ation reaction was carried out on (I) using CH_3_I, resulting in compound (II).
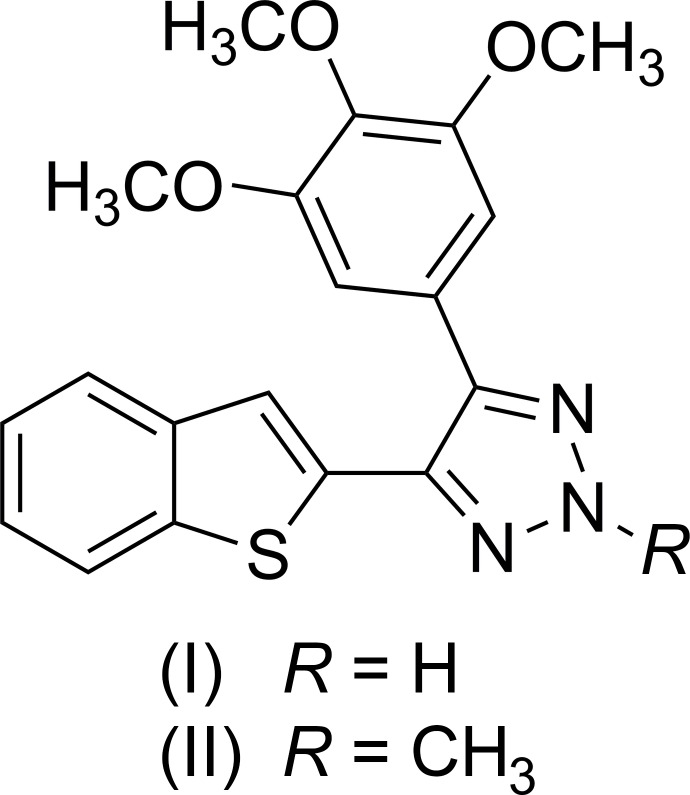


## Structural commentary   

**Figure 1:**
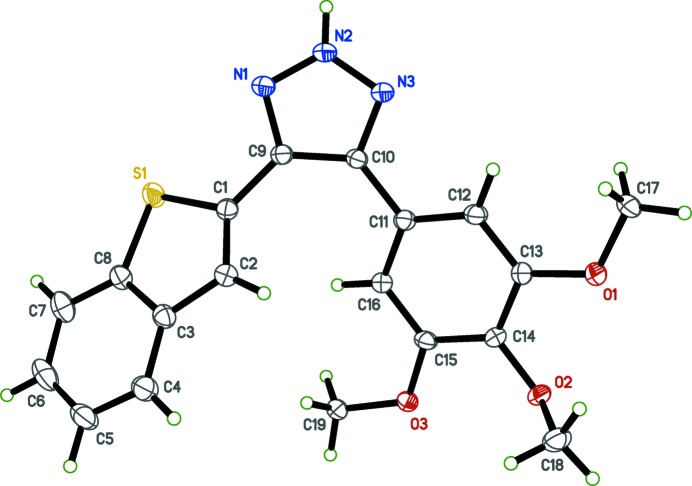
The mol­ecular structure of (I), with displacement ellipsoids drawn at the 50% probability level.

In order to obtain detailed information on the structural conformations of (I) and (II) for analysis of structure–activity relationships (SAR), including the position of the hydrogen atom in the triazole ring system of (I) and the position of methyl­ation on the triazole ring system in (II), we determined the X-ray crystal structures of (I) and (II); see Figs. 1 ▶ and 2 ▶, respectively.

Selected geometric parameters are given in Tables 1 ▶ and 2 ▶ for (I) and (II), respectively. The benzo­thio­phene rings are almost planar with r.m.s deviations from the mean plane of 0.0205 (14) in (I) and 0.016 (2) Å in (II), with bond distances and angles comparable with those reported for other benzo­thio­phene derivatives (Sonar *et al.*, 2007 ▶) and triazole analogs (Madadi *et al.*, 2014 ▶). The triazole rings make dihedral angles of 32.68 (5)° and 10.43 (8)°, respectively, in (I) and (II) with the mean plane of the benzo­thio­phene ring systems. The tri­meth­oxy­phenyl rings make dihedral angles of 38.48 (4) in (I) and 60.43 (5)° in (II) with the benzo­thio­phene ring systems. In both compounds (I) and (II), deviations from ideal geometry are observed in the bond angles C1—S1—C8, N2—N1—C9, N2—N3—C10, which are compressed, and C1—C9—C10, C9—C10—C11, C2—C3—C4, which are expanded (see Tables 1 ▶ and 2 ▶). After *N*-methyl­ation, no significant difference is observed for the N1—N2—N3 bond angle [116.2 (1) and 115.9 (1)°, respectively, for (I) and (II)]. The crystal structure of (II) has a minor component of disorder that corresponds to a 180° flip of the benzo­thio­phene ring system [occupancy ratio 0.9363 (14):0.0637 (14)].

## Supra­molecular features   

**Figure 3:**
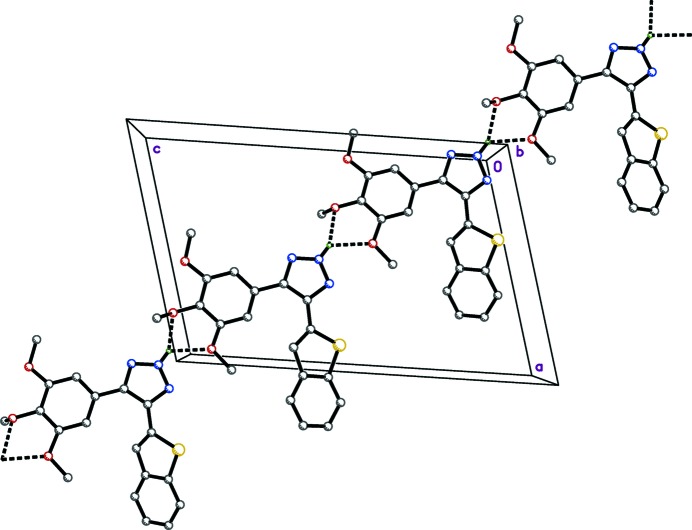
Hydrogen bonding in the crystal structure of (I), viewed along the *b* axis. Dashed lines represent hydrogen bonds, which join mol­ecules into chains along the [101] direction.

**Figure 4:**
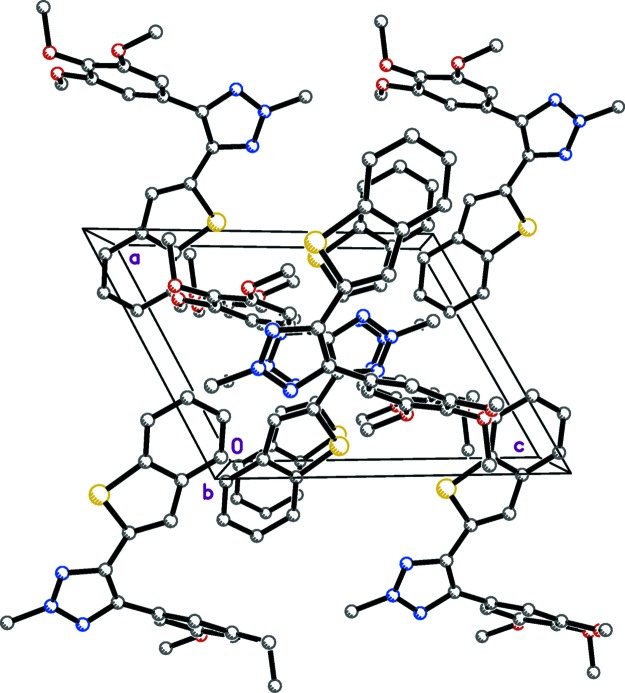
Crystal packing of (II), as viewed along the *b* axis.

Hydrogen bonding and the mode of packing of (I) is illus­trated in Fig. 3 ▶, and the mode of packing of (II) is illustrated in Fig. 4 ▶. In the structure of (I), the mol­ecules are linked by inter­molecular hydrogen bonds (N2—H2*N*⋯O2 and N2—H2*N*⋯O3), forming 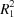
(5) ring motifs (Table 3 ▶), which propagate as chains along the [101] direction. Contacts between adjacent chains form two-dimensional pleated-sheet networks in the *ac* plane. No significant hydrogen-bonding inter­actions were found in the structure of (II).

## Database survey   

A search of the 2014 release of the Cambridge Structural Database on unit-cell dimensions for (I) and (II) revealed four triazole structures (HOZZAY, UPEWAO, SAFZEG & VUSNEC), although none bore any particular relation to compounds (I) or (II). A search on the triazole ring fragment with either H or methyl attached to the middle N atom revealed 48 and 17 hits, respectively, none of which contained either benzo­thio­phene or tri­meth­oxy­benzene functional groups.

## Synthesis and crystallization   

The title compounds were prepared according to a previously reported procedure (Penthala *et al.*, 2014 ▶). Recrystallization from methanol afforded (I) and (II) as yellow and pale-yellow crystalline products, respectively, which were suitable for X-ray analysis.

## Refinement details   

Crystal data, data collection and structure refinement details are summarized in Table 4 ▶. H atoms were found in difference Fourier maps. Carbon-bound hydrogens were subsequently placed at idealized positions with constrained distances of 0.98 (*R*CH_3_) and 0.95 Å (C*sp* ^2^H). Coordinates of the N-bound hydrogen were refined freely. *U*_iso_(H) values were set to either 1.2*U*_eq_ or 1.5*U*_eq_ (*R*CH_3_) of the attached atom.

Refinement progress was checked using *PLATON* (Spek, 2009 ▶) and by an *R*-tensor (Parkin, 2000 ▶). To ensure satisfactory refinement of disordered groups in the structure, a combination of constraints and restraints was employed. The constraints (*SHELXL* command EADP) were used to fix overlapping fragments. Restraints were used to maintain the integrity of ill-defined or disordered groups (*SHELXL* commands SAME and RIGU).

In structure (II), there was a small amount of a second conformation for the benzo­thio­phene ring systems, with major and minor component fractions of 93.63 (14) and 6.37 (14)%, respectively.

## Supplementary Material

Crystal structure: contains datablock(s) global, I, II. DOI: 10.1107/S1600536814023095/hg5414sup1.cif

Structure factors: contains datablock(s) I. DOI: 10.1107/S1600536814023095/hg5414Isup2.hkl

Structure factors: contains datablock(s) II. DOI: 10.1107/S1600536814023095/hg5414IIsup3.hkl

Supporting information file. DOI: 10.1107/S1600536814023095/hg5414Isup4.cml

Supporting information file. DOI: 10.1107/S1600536814023095/hg5414IIsup5.cml

CCDC references: 1030172, 1030173

Additional supporting information:  crystallographic information; 3D view; checkCIF report

## Figures and Tables

**Figure 2:**
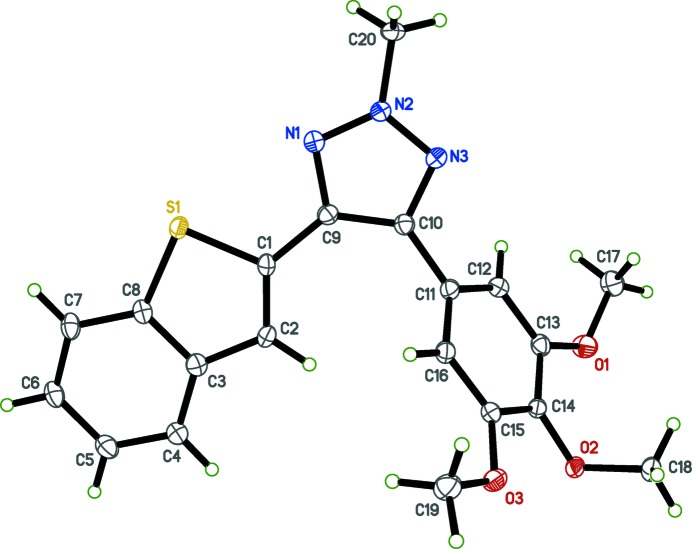
The mol­ecular structure of (II), with displacement ellipsoids drawn at the 50% probability level.

**Table 1:** Selected geometric parameters (, ) for (I)

N1N2	1.324(2)	N2H2*N*	0.87(2)
N1C9	1.343(2)	N3C10	1.345(2)
N2N3	1.330(2)		
			
C8S1C1	91.50(8)	C10C9C1	131.64(14)
N2N1C9	103.74(13)	C9C10C11	131.16(14)
N2N3C10	103.74(13)	O1C13C14	114.89(14)
C4C3C2	129.50(16)		

**Table 2:** Selected geometric parameters (, ) for (II)

N1N2	1.3266(15)	N2C20	1.4527(16)
N1C9	1.3477(16)	N3C10	1.3450(16)
N2N3	1.3279(15)		
			
N1N2N3	115.92(10)	C4C3C2	132(2)
C2C1C9	129.94(17)	C7C8S1	129(2)
C8S1C1	91.33(8)	C1C9C10	127.3(11)
C4C3C2	129.79(17)	C10C9C1	132.41(13)
C9C1S1	128.0(18)	C9C10C11	132.90(12)
C8S1C1	95.8(12)		

**Table 3:** Hydrogen-bond geometry (, ) for (I)

*D*H*A*	*D*H	H*A*	*D* *A*	*D*H*A*
N2H2*N*O2^i^	0.87(2)	2.16(2)	2.9381(18)	147.6(18)
N2H2*N*O3^i^	0.87(2)	2.20(2)	2.8503(18)	130.8(17)

Symmetry code: (i) 
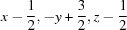
.

**Table 4:** Experimental details

	(I)	(II)
Crystal data		
Chemical formula	C_19_H_17_N_3_O_3_S	C_20_H_19_N_3_O_3_S
*M*_r_	367.41	381.44
Crystal system, space group	Monoclinic, *P*2_1_/*n*	Triclinic, *P* 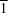
Temperature (K)	90	90
*a*, *b*, *c* ()	11.8983(2), 8.1860(1), 18.4582(3)	8.8579(1), 11.0761(1), 11.2626(1)
, , ()	90, 105.5046(7), 90	106.859(4), 111.668(5), 105.498(4)
*V* (^3^)	1732.39(5)	891.51(4)
*Z*	4	2
Radiation type	Mo *K*	Mo *K*
(mm^1^)	0.21	0.21
Crystal size (mm)	0.30 0.30 0.05	0.22 0.20 0.15

Data collection		
Diffractometer	Nonius KappaCCD	Nonius KappaCCD
Absorption correction	Multi-scan (*SADABS*; Sheldrick, 2008*a* ▶)	Multi-scan (*SADABS*; Sheldrick, 2008*a* ▶)
*T*_min_, *T*_max_	0.816, 0.966	0.858, 0.962
No. of measured, independent and observed [*I* > 2(*I*)] reflections	28105, 3984, 3093	36591, 4097, 3572
*R*_int_	0.045	0.045
(sin /)_max_ (^1^)	0.650	0.651

Refinement		
*R*[*F* ^2^ > 2(*F* ^2^)], *wR*(*F* ^2^), *S*	0.044, 0.124, 1.07	0.037, 0.096, 1.08
No. of reflections	3984	4097
No. of parameters	241	276
No. of restraints	0	161
H-atom treatment	H atoms treated by a mixture of independent and constrained refinement	H-atom parameters constrained
_max_, _min_ (e ^3^)	0.55, 0.29	0.31, 0.28

Computer programs: *COLLECT* (Nonius, 1998 ▶), *SCALEPACK* and *DENZO-SMN* (Otwinowski Minor, 2006 ▶), *SHELXS97*, *SHELXL2013*, *SHELXL2014* and *XP* in *SHELXTL* (Sheldrick, 2008*b* ▶) and *CIFFIX* (Parkin, 2013 ▶).

## supplementary crystallographic information

### Crystal data

**Table:**

C_20_H_19_N_3_O_3_S	*Z* = 2
*M**_r_* = 381.44	*F*(000) = 400
Triclinic, *P*1	*D*_x_ = 1.421 Mg m^−^^3^
*a* = 8.8579 (1) Å	Mo *K*α radiation, λ = 0.71073 Å
*b* = 11.0761 (1) Å	Cell parameters from 4076 reflections
*c* = 11.2626 (1) Å	θ = 1.0–27.5°
α = 106.859 (4)°	µ = 0.21 mm^−^^1^
β = 111.668 (5)°	*T* = 90 K
γ = 105.498 (4)°	Cut block, pale yellow
*V* = 891.51 (4) Å^3^	0.22 × 0.20 × 0.15 mm

### Data collection

**Table:**

Nonius KappaCCD diffractometer	4097 independent reflections
Radiation source: fine-focus sealed-tube	3572 reflections with *I* > 2σ(*I*)
Detector resolution: 9.1 pixels mm^-1^	*R*_int_ = 0.045
φ and ω scans at fixed χ = 55°	θ_max_ = 27.6°, θ_min_ = 2.1°
Absorption correction: multi-scan (*SADABS*; Sheldrick, 2008a)	*h* = −11→11
*T*_min_ = 0.858, *T*_max_ = 0.962	*k* = −14→14
36591 measured reflections	*l* = −14→14

### Refinement

**Table:**

Refinement on *F*^2^	Primary atom site location: structure-invariant direct methods
Least-squares matrix: full	Secondary atom site location: difference Fourier map
*R*[*F*^2^ > 2σ(*F*^2^)] = 0.037	Hydrogen site location: difference Fourier map
*wR*(*F*^2^) = 0.096	H-atom parameters constrained
*S* = 1.08	*w* = 1/[σ^2^(*F*_o_^2^) + (0.0472*P*)^2^ + 0.4023*P*] where *P* = (*F*_o_^2^ + 2*F*_c_^2^)/3
4097 reflections	(Δ/σ)_max_ = 0.001
276 parameters	Δρ_max_ = 0.31 e Å^−^^3^
161 restraints	Δρ_min_ = −0.28 e Å^−^^3^

### Special details

**Table:**

Experimental. The crystal was mounted with polyisobutene oil on the tip of a fine glass fibre, which was fastened in a copper mounting pin with electrical solder. It was placed directly into the cold gas stream of a liquid nitrogen based cryostat, according to published methods (Hope, 1994; Parkin & Hope, 1998).Diffraction data were collected with the crystal at 90 K, which is standard practice in this laboratory for the majority of flash-cooled crystals.
Geometry. All e.s.d.'s (except the e.s.d. in the dihedral angle between two l.s. planes) are estimated using the full covariance matrix. The cell e.s.d.'s are taken into account individually in the estimation of e.s.d.'s in distances, angles and torsion angles; correlations between e.s.d.'s in cell parameters are only used when they are defined by crystal symmetry. An approximate (isotropic) treatment of cell e.s.d.'s is used for estimating e.s.d.'s involving l.s. planes.
Refinement. Refinement progress was checked using *PLATON* (Spek, 2009) and by an *R*-tensor (Parkin, 2000). The final model was further checked with the IUCr utility *checkCIF*.

### Fractional atomic coordinates and isotropic or equivalent isotropic displacement parameters (Å^2^)

**Table:**

	*x*	*y*	*z*	*U*_iso_*/*U*_eq_	Occ. (<1)
N1	0.41837 (14)	0.62237 (11)	0.61716 (11)	0.0160 (2)	
N2	0.56418 (15)	0.73837 (11)	0.71234 (11)	0.0158 (2)	
N3	0.65185 (15)	0.80574 (11)	0.66113 (11)	0.0163 (2)	
O1	0.75768 (14)	1.08896 (10)	0.37490 (10)	0.0234 (2)	
O2	0.77188 (12)	0.90655 (10)	0.16639 (9)	0.0178 (2)	
O3	0.72458 (13)	0.64975 (10)	0.14267 (10)	0.0197 (2)	
C1	0.2506 (5)	0.5036 (4)	0.36036 (19)	0.0145 (4)	0.9363 (14)
S1	0.08551 (5)	0.39081 (4)	0.37446 (4)	0.01741 (12)	0.9363 (14)
C2	0.2034 (2)	0.48053 (18)	0.22483 (17)	0.0176 (3)	0.9363 (14)
H2	0.2776	0.5336	0.1986	0.021*	0.9363 (14)
C3	0.0313 (3)	0.3687 (3)	0.1242 (2)	0.0164 (4)	0.9363 (14)
C4	−0.0638 (3)	0.31782 (16)	−0.0245 (2)	0.0203 (4)	0.9363 (14)
H4	−0.0117	0.3565	−0.0725	0.024*	0.9363 (14)
C5	−0.2341 (3)	0.21078 (18)	−0.09952 (19)	0.0210 (4)	0.9363 (14)
H5	−0.2990	0.1766	−0.1996	0.025*	0.9363 (14)
C6	−0.3125 (2)	0.1519 (2)	−0.03024 (16)	0.0195 (4)	0.9363 (14)
H6	−0.4295	0.0784	−0.0841	0.023*	0.9363 (14)
C7	−0.2218 (2)	0.19944 (19)	0.11543 (18)	0.0186 (4)	0.9363 (14)
H7	−0.2742	0.1591	0.1624	0.022*	0.9363 (14)
C8	−0.0508 (2)	0.3086 (2)	0.19147 (18)	0.0161 (3)	0.9363 (14)
C1'	0.269 (8)	0.522 (7)	0.368 (2)	0.0145 (4)	0.0637 (14)
S1'	0.2341 (9)	0.5088 (7)	0.2013 (7)	0.0176 (3)	0.0637 (14)
C2'	0.134 (3)	0.418 (2)	0.3485 (19)	0.01741 (12)	0.0637 (14)
H2'	0.1347	0.3980	0.4251	0.021*	0.0637 (14)
C3'	−0.013 (4)	0.335 (4)	0.207 (2)	0.0161 (3)	0.0637 (14)
C4'	−0.173 (3)	0.221 (3)	0.150 (3)	0.0186 (4)	0.0637 (14)
H4'	−0.1949	0.1828	0.2106	0.022*	0.0637 (14)
C5'	−0.297 (4)	0.162 (3)	0.013 (3)	0.0195 (4)	0.0637 (14)
H5'	−0.4184	0.1070	−0.0167	0.023*	0.0637 (14)
C6'	−0.248 (4)	0.183 (3)	−0.086 (3)	0.0210 (4)	0.0637 (14)
H6'	−0.3283	0.1303	−0.1851	0.025*	0.0637 (14)
C7'	−0.079 (4)	0.284 (3)	−0.036 (3)	0.0203 (4)	0.0637 (14)
H7'	−0.0314	0.2892	−0.0985	0.024*	0.0637 (14)
C8'	0.020 (4)	0.377 (5)	0.108 (2)	0.0164 (4)	0.0637 (14)
C9	0.40890 (17)	0.61277 (13)	0.49233 (13)	0.0147 (2)	
C10	0.55623 (17)	0.72729 (13)	0.52005 (13)	0.0146 (2)	
C11	0.61431 (17)	0.77268 (13)	0.42743 (13)	0.0152 (3)	
C12	0.65731 (17)	0.91063 (13)	0.44855 (13)	0.0166 (3)	
H12	0.6472	0.9730	0.5204	0.020*	
C13	0.71519 (17)	0.95607 (13)	0.36342 (14)	0.0168 (3)	
C14	0.72907 (16)	0.86471 (13)	0.25757 (13)	0.0152 (3)	
C15	0.69315 (17)	0.72810 (13)	0.24151 (13)	0.0157 (3)	
C16	0.63395 (17)	0.68128 (13)	0.32534 (13)	0.0158 (3)	
H16	0.6073	0.5881	0.3131	0.019*	
C17	0.8251 (2)	1.19553 (14)	0.51199 (15)	0.0231 (3)	
H17A	0.7287	1.1871	0.5366	0.035*	
H17B	0.8707	1.2866	0.5122	0.035*	
H17C	0.9222	1.1864	0.5817	0.035*	
C18	0.96108 (18)	0.98269 (14)	0.22249 (14)	0.0202 (3)	
H18A	1.0093	1.0624	0.3134	0.030*	
H18B	0.9813	1.0158	0.1555	0.030*	
H18C	1.0214	0.9217	0.2368	0.030*	
C19	0.7008 (2)	0.51278 (14)	0.13104 (16)	0.0231 (3)	
H19A	0.7781	0.5191	0.2233	0.035*	
H19B	0.7324	0.4680	0.0608	0.035*	
H19C	0.5754	0.4577	0.1010	0.035*	
C20	0.63205 (18)	0.78558 (14)	0.86407 (13)	0.0185 (3)	
H20A	0.7016	0.7356	0.8977	0.028*	
H20B	0.5314	0.7673	0.8838	0.028*	
H20C	0.7089	0.8856	0.9131	0.028*	

### Atomic displacement parameters (Å^2^)

**Table:**

	*U*^11^	*U*^22^	*U*^33^	*U*^12^	*U*^13^	*U*^23^
N1	0.0151 (5)	0.0152 (5)	0.0154 (5)	0.0047 (4)	0.0069 (4)	0.0059 (4)
N2	0.0165 (5)	0.0154 (5)	0.0138 (5)	0.0042 (4)	0.0078 (4)	0.0059 (4)
N3	0.0167 (5)	0.0171 (5)	0.0156 (5)	0.0054 (4)	0.0087 (4)	0.0080 (4)
O1	0.0339 (6)	0.0148 (5)	0.0214 (5)	0.0074 (4)	0.0139 (4)	0.0095 (4)
O2	0.0152 (5)	0.0207 (5)	0.0139 (4)	0.0021 (4)	0.0061 (4)	0.0093 (4)
O3	0.0235 (5)	0.0176 (5)	0.0206 (5)	0.0070 (4)	0.0147 (4)	0.0077 (4)
C1	0.0134 (11)	0.0112 (15)	0.0170 (6)	0.0034 (6)	0.0072 (6)	0.0057 (6)
S1	0.01419 (19)	0.01807 (19)	0.01687 (18)	0.00243 (14)	0.00642 (14)	0.00913 (14)
C2	0.0158 (7)	0.0180 (8)	0.0178 (7)	0.0032 (6)	0.0091 (6)	0.0086 (6)
C3	0.0152 (7)	0.0161 (7)	0.0178 (7)	0.0070 (6)	0.0078 (6)	0.0069 (6)
C4	0.0197 (8)	0.0183 (10)	0.0186 (7)	0.0045 (8)	0.0098 (6)	0.0051 (7)
C5	0.0192 (7)	0.0194 (9)	0.0169 (7)	0.0056 (7)	0.0054 (6)	0.0051 (6)
C6	0.0139 (7)	0.0159 (7)	0.0197 (9)	0.0032 (5)	0.0030 (7)	0.0059 (8)
C7	0.0123 (8)	0.0167 (8)	0.0219 (10)	0.0039 (7)	0.0043 (7)	0.0096 (8)
C8	0.0138 (10)	0.0135 (11)	0.0179 (7)	0.0048 (7)	0.0057 (6)	0.0062 (7)
C1'	0.0134 (11)	0.0112 (15)	0.0170 (6)	0.0034 (6)	0.0072 (6)	0.0057 (6)
S1'	0.0158 (7)	0.0180 (8)	0.0178 (7)	0.0032 (6)	0.0091 (6)	0.0086 (6)
C2'	0.01419 (19)	0.01807 (19)	0.01687 (18)	0.00243 (14)	0.00642 (14)	0.00913 (14)
C3'	0.0138 (10)	0.0135 (11)	0.0179 (7)	0.0048 (7)	0.0057 (6)	0.0062 (7)
C4'	0.0123 (8)	0.0167 (8)	0.0219 (10)	0.0039 (7)	0.0043 (7)	0.0096 (8)
C5'	0.0139 (7)	0.0159 (7)	0.0197 (9)	0.0032 (5)	0.0030 (7)	0.0059 (8)
C6'	0.0192 (7)	0.0194 (9)	0.0169 (7)	0.0056 (7)	0.0054 (6)	0.0051 (6)
C7'	0.0197 (8)	0.0183 (10)	0.0186 (7)	0.0045 (8)	0.0098 (6)	0.0051 (7)
C8'	0.0152 (7)	0.0161 (7)	0.0178 (7)	0.0070 (6)	0.0078 (6)	0.0069 (6)
C9	0.0155 (6)	0.0149 (6)	0.0143 (6)	0.0065 (5)	0.0072 (5)	0.0073 (5)
C10	0.0139 (6)	0.0148 (6)	0.0145 (6)	0.0059 (5)	0.0067 (5)	0.0062 (5)
C11	0.0129 (6)	0.0168 (6)	0.0139 (6)	0.0042 (5)	0.0055 (5)	0.0074 (5)
C12	0.0168 (6)	0.0161 (6)	0.0143 (6)	0.0053 (5)	0.0070 (5)	0.0057 (5)
C13	0.0161 (6)	0.0145 (6)	0.0161 (6)	0.0038 (5)	0.0055 (5)	0.0075 (5)
C14	0.0122 (6)	0.0179 (6)	0.0123 (6)	0.0027 (5)	0.0045 (5)	0.0076 (5)
C15	0.0126 (6)	0.0171 (6)	0.0129 (6)	0.0039 (5)	0.0049 (5)	0.0048 (5)
C16	0.0144 (6)	0.0149 (6)	0.0162 (6)	0.0040 (5)	0.0068 (5)	0.0070 (5)
C17	0.0271 (7)	0.0144 (6)	0.0252 (7)	0.0079 (6)	0.0121 (6)	0.0066 (6)
C18	0.0161 (6)	0.0214 (7)	0.0201 (7)	0.0034 (5)	0.0086 (5)	0.0094 (6)
C19	0.0295 (8)	0.0184 (7)	0.0257 (7)	0.0106 (6)	0.0178 (6)	0.0085 (6)
C20	0.0225 (7)	0.0193 (6)	0.0126 (6)	0.0070 (5)	0.0089 (5)	0.0063 (5)

### Geometric parameters (Å, º)

**Table:**

N1—N2	1.3266 (15)	C3'—C4'	1.383 (17)
N1—C9	1.3477 (16)	C3'—C8'	1.413 (17)
N2—N3	1.3279 (15)	C4'—C5'	1.346 (17)
N2—C20	1.4527 (16)	C4'—H4'	0.9500
N3—C10	1.3450 (16)	C5'—C6'	1.393 (18)
O1—C13	1.3720 (15)	C5'—H5'	0.9500
O1—C17	1.4210 (17)	C6'—C7'	1.385 (18)
O2—C14	1.3721 (14)	C6'—H6'	0.9500
O2—C18	1.4398 (16)	C7'—C8'	1.405 (18)
O3—C15	1.3666 (15)	C7'—H7'	0.9500
O3—C19	1.4340 (16)	C9—C10	1.4122 (17)
C1—C2	1.347 (3)	C10—C11	1.4723 (17)
C1—C9	1.466 (2)	C11—C16	1.3943 (18)
C1—S1	1.742 (2)	C11—C12	1.3956 (18)
S1—C8	1.7380 (17)	C12—C13	1.3930 (18)
C2—C3	1.429 (2)	C12—H12	0.9500
C2—H2	0.9500	C13—C14	1.3911 (18)
C3—C8	1.409 (2)	C14—C15	1.4018 (18)
C3—C4	1.410 (2)	C15—C16	1.3926 (17)
C4—C5	1.384 (2)	C16—H16	0.9500
C4—H4	0.9500	C17—H17A	0.9800
C5—C6	1.404 (2)	C17—H17B	0.9800
C5—H5	0.9500	C17—H17C	0.9800
C6—C7	1.383 (2)	C18—H18A	0.9800
C6—H6	0.9500	C18—H18B	0.9800
C7—C8	1.397 (2)	C18—H18C	0.9800
C7—H7	0.9500	C19—H19A	0.9800
C1'—C2'	1.318 (19)	C19—H19B	0.9800
C1'—C9	1.32 (2)	C19—H19C	0.9800
C1'—S1'	1.74 (2)	C20—H20A	0.9800
S1'—C8'	1.731 (18)	C20—H20B	0.9800
C2'—C3'	1.439 (17)	C20—H20C	0.9800
C2'—H2'	0.9500		
			
N2—N1—C9	103.78 (10)	C8'—C7'—H7'	120.8
N1—N2—N3	115.92 (10)	C7'—C8'—C3'	119 (2)
N1—N2—C20	122.69 (11)	C7'—C8'—S1'	129 (2)
N3—N2—C20	121.27 (11)	C3'—C8'—S1'	106.9 (14)
N2—N3—C10	104.04 (10)	C1'—C9—N1	123.7 (13)
C13—O1—C17	116.24 (10)	C1'—C9—C10	127.3 (11)
C14—O2—C18	113.82 (10)	N1—C9—C10	108.26 (11)
C15—O3—C19	116.41 (10)	N1—C9—C1	118.94 (13)
C2—C1—C9	129.94 (17)	C10—C9—C1	132.41 (13)
C2—C1—S1	112.33 (14)	N3—C10—C9	108.00 (11)
C9—C1—S1	117.55 (13)	N3—C10—C11	119.08 (11)
C8—S1—C1	91.33 (8)	C9—C10—C11	132.90 (12)
C1—C2—C3	113.73 (19)	C16—C11—C12	120.79 (11)
C1—C2—H2	123.1	C16—C11—C10	120.52 (11)
C3—C2—H2	123.1	C12—C11—C10	118.61 (11)
C8—C3—C4	118.66 (16)	C13—C12—C11	119.45 (12)
C8—C3—C2	111.50 (15)	C13—C12—H12	120.3
C4—C3—C2	129.79 (17)	C11—C12—H12	120.3
C5—C4—C3	119.15 (16)	O1—C13—C14	116.02 (11)
C5—C4—H4	120.4	O1—C13—C12	123.56 (12)
C3—C4—H4	120.4	C14—C13—C12	120.40 (12)
C4—C5—C6	121.14 (16)	O2—C14—C13	120.25 (11)
C4—C5—H5	119.4	O2—C14—C15	120.12 (11)
C6—C5—H5	119.4	C13—C14—C15	119.60 (11)
C7—C6—C5	120.88 (16)	O3—C15—C16	124.29 (12)
C7—C6—H6	119.6	O3—C15—C14	115.29 (11)
C5—C6—H6	119.6	C16—C15—C14	120.40 (12)
C6—C7—C8	118.04 (16)	C15—C16—C11	119.24 (12)
C6—C7—H7	121.0	C15—C16—H16	120.4
C8—C7—H7	121.0	C11—C16—H16	120.4
C7—C8—C3	122.13 (15)	O1—C17—H17A	109.5
C7—C8—S1	126.75 (13)	O1—C17—H17B	109.5
C3—C8—S1	111.11 (11)	H17A—C17—H17B	109.5
C2'—C1'—C9	124.6 (18)	O1—C17—H17C	109.5
C2'—C1'—S1'	107.3 (15)	H17A—C17—H17C	109.5
C9—C1'—S1'	128.0 (18)	H17B—C17—H17C	109.5
C8'—S1'—C1'	95.8 (12)	O2—C18—H18A	109.5
C1'—C2'—C3'	117.3 (18)	O2—C18—H18B	109.5
C1'—C2'—H2'	121.4	H18A—C18—H18B	109.5
C3'—C2'—H2'	121.4	O2—C18—H18C	109.5
C4'—C3'—C8'	115.8 (17)	H18A—C18—H18C	109.5
C4'—C3'—C2'	132 (2)	H18B—C18—H18C	109.5
C8'—C3'—C2'	111.8 (16)	O3—C19—H19A	109.5
C5'—C4'—C3'	122 (2)	O3—C19—H19B	109.5
C5'—C4'—H4'	118.9	H19A—C19—H19B	109.5
C3'—C4'—H4'	118.9	O3—C19—H19C	109.5
C4'—C5'—C6'	120 (2)	H19A—C19—H19C	109.5
C4'—C5'—H5'	120.2	H19B—C19—H19C	109.5
C6'—C5'—H5'	120.2	N2—C20—H20A	109.5
C7'—C6'—C5'	118 (2)	N2—C20—H20B	109.5
C7'—C6'—H6'	121.0	H20A—C20—H20B	109.5
C5'—C6'—H6'	121.0	N2—C20—H20C	109.5
C6'—C7'—C8'	118 (2)	H20A—C20—H20C	109.5
C6'—C7'—H7'	120.8	H20B—C20—H20C	109.5
			
C9—N1—N2—N3	−0.21 (14)	S1'—C1'—C9—C10	6 (12)
C9—N1—N2—C20	−176.39 (11)	S1'—C1'—C9—C1	−157 (59)
N1—N2—N3—C10	−0.26 (14)	N2—N1—C9—C1'	−170 (6)
C20—N2—N3—C10	175.99 (11)	N2—N1—C9—C10	0.57 (13)
C2—C1—S1—C8	0.6 (4)	N2—N1—C9—C1	−173.1 (3)
C9—C1—S1—C8	176.2 (4)	S1—C1—C9—C1'	−154 (51)
C9—C1—C2—C3	−175.7 (5)	C2—C1—C9—N1	173.7 (5)
S1—C1—C2—C3	−0.7 (5)	S1—C1—C9—N1	−1.1 (5)
C1—C2—C3—C8	0.5 (4)	C2—C1—C9—C10	1.8 (8)
C1—C2—C3—C4	177.8 (4)	S1—C1—C9—C10	−172.95 (18)
C8—C3—C4—C5	0.0 (4)	N2—N3—C10—C9	0.60 (13)
C2—C3—C4—C5	−177.2 (3)	N2—N3—C10—C11	179.11 (11)
C3—C4—C5—C6	−0.4 (3)	C1'—C9—C10—N3	170 (6)
C4—C5—C6—C7	0.1 (3)	N1—C9—C10—N3	−0.76 (14)
C5—C6—C7—C8	0.6 (3)	C1—C9—C10—N3	171.7 (4)
C6—C7—C8—C3	−1.1 (4)	C1'—C9—C10—C11	−9 (6)
C6—C7—C8—S1	177.16 (19)	N1—C9—C10—C11	−178.99 (13)
C4—C3—C8—C7	0.8 (4)	C1—C9—C10—C11	−6.5 (4)
C2—C3—C8—C7	178.4 (2)	N3—C10—C11—C16	129.29 (13)
C4—C3—C8—S1	−177.7 (2)	C9—C10—C11—C16	−52.6 (2)
C2—C3—C8—S1	−0.1 (3)	N3—C10—C11—C12	−47.72 (17)
C1—S1—C8—C7	−178.7 (3)	C9—C10—C11—C12	130.36 (15)
C1—S1—C8—C3	−0.3 (3)	C16—C11—C12—C13	2.02 (19)
C2'—C1'—S1'—C8'	9 (7)	C10—C11—C12—C13	179.01 (12)
C9—C1'—S1'—C8'	−176 (9)	C17—O1—C13—C14	−151.61 (12)
C9—C1'—C2'—C3'	175 (7)	C17—O1—C13—C12	29.78 (18)
S1'—C1'—C2'—C3'	−8 (8)	C11—C12—C13—O1	178.95 (12)
C1'—C2'—C3'—C4'	−180 (7)	C11—C12—C13—C14	0.40 (19)
C1'—C2'—C3'—C8'	4 (8)	C18—O2—C14—C13	82.87 (15)
C8'—C3'—C4'—C5'	−7 (7)	C18—O2—C14—C15	−99.29 (14)
C2'—C3'—C4'—C5'	177 (4)	O1—C13—C14—O2	−4.02 (18)
C3'—C4'—C5'—C6'	21 (6)	C12—C13—C14—O2	174.63 (11)
C4'—C5'—C6'—C7'	−11 (6)	O1—C13—C14—C15	178.13 (11)
C5'—C6'—C7'—C8'	−12 (7)	C12—C13—C14—C15	−3.22 (19)
C6'—C7'—C8'—C3'	27 (8)	C19—O3—C15—C16	−2.60 (18)
C6'—C7'—C8'—S1'	178 (4)	C19—O3—C15—C14	175.84 (11)
C4'—C3'—C8'—C7'	−17 (8)	O2—C14—C15—O3	7.32 (17)
C2'—C3'—C8'—C7'	160 (5)	C13—C14—C15—O3	−174.83 (11)
C4'—C3'—C8'—S1'	−174 (4)	O2—C14—C15—C16	−174.18 (11)
C2'—C3'—C8'—S1'	3 (6)	C13—C14—C15—C16	3.68 (19)
C1'—S1'—C8'—C7'	−160 (6)	O3—C15—C16—C11	177.06 (12)
C1'—S1'—C8'—C3'	−6 (6)	C14—C15—C16—C11	−1.30 (19)
C2'—C1'—C9—N1	−10 (12)	C12—C11—C16—C15	−1.56 (19)
S1'—C1'—C9—N1	174 (5)	C10—C11—C16—C15	−178.49 (12)
C2'—C1'—C9—C10	−179 (6)		
